# Corrosion Characteristics of Rolling Oil on the Rolled Copper Foil

**DOI:** 10.3390/ma13214933

**Published:** 2020-11-03

**Authors:** Lei Xia, Yan Li, Shen Zhao, Sang Xiong, Zhengyi Jiang

**Affiliations:** 1 School of Material and Metallurgy, University of Science and Technology Liaoning, Anshan 114051, China; xialei0627@163.com; 2 Ansteel Iron & Steel Research Institute, Ansteel Group Corporation, Anshan 114009, China; 3 Research & Development Department, Nanjing MDP Technology Co., Ltd., Nanjing 211300, China; zhaoshenzhsh@163.com; 4 College of Materials Engineering, Nanjing Institute of Technology, Nanjing 211167, China; wb9201@126.com

**Keywords:** corrosion, rolled copper foil, rolling oil, friction modifier, extreme pressure additives

## Abstract

Static corrosion experiments were carried out to investigate the corrosion of each kind of component in the rolling oil on the rolled copper foil. The surface morphology and chemical composition of corrosion products were detected by a digital camera, scanning electron microscope (SEM), energy dispersive spectrometer (EDS) and X-ray photoelectron spectroscopy (XPS). The results showed that the maximum corrosion rate of rolled copper foil in the base stock and friction modifiers (butyl stearate and dodecanol) was close to zero, while that of rolled copper foil in the N-containing borate, phosphate and the fully formulated rolling oil were 0.17, 1.12 and 0.78 mm/a, respectively. The color of rolled copper foil changing from pink into purple-black when corroded in the N-containing borate. The composition of it was mainly CuO and Cu_2_O with some N-containing borate adsorbed on it. However, the color and composition of the corroded copper foil in the phosphate were similar to that of the original copper foil. It was complicated for the corroded copper foil in the fully formulated rolling oil, which showed characteristics both in the N-containing borate and in the phosphate according to different positions. It indicated that there might be little corrosion for the base stock and friction modifiers on the rolled copper foil. It might mainly be extreme pressure additives (N-containing borate and phosphate) that caused the corrosion of rolled copper foil. There might be competition between N-containing borate and phosphate for the corrosion of rolled copper foil in the fully formulated rolling oil, resulting in a lower corrosion rate compared with that in the phosphate.

## 1. Introduction

With the rapid development of the electronic information industry, more and more attention has been paid to the rolled copper foil due to its high quality and high reliability [1,2]. As a widely used and important non-ferrous metal material, the lubrication in the production process has been of wide concern in order to produce a rolled copper foil with excellent properties. The application of lubrication technology in the production process of rolled copper foil can effectively reduce the minimum rolling thickness, improve flatness, and decrease energy consumption [3,4]. However, the rolling oil used in lubrication may cause corrosion to the surface of rolled copper foil and then affect the quality of rolled copper foil.

For a long time, the oxidation discoloration problem of copper has been puzzling in the production of copper and copper alloys [5]. It has become an important factor affecting the quality of copper, especially for rolled copper foil with high dimensional accuracy. Therefore, the rolling oil used in lubrication should not lead to corrosion on the surface of rolled copper foil, in addition to excellent lubricity. It is always contradictory between improving the lubricity of rolling oil and reducing the corrosion of rolling oil on the rolled copper foil. Functional additives, such as friction modifiers, extreme pressure agents, are often added into the rolling oil to improve its lubricating performance [6,7,8]. Some of them work by reacting with the rolled copper foil, which may cause the corrosion of rolled copper foil and affect its surface quality.

Adding a certain amount of friction modifiers into the rolling oil can improve its lubricating performance under mild conditions. Fatty alcohol, fatty acid and fatty acid ester are commonly used friction modifiers in oil-based lubricants [9,10]. The lubricating performance of rolling oil can be improved by extreme pressure agents under severe conditions, which is usually achieved by forming a film with better anti-friction and anti-wear performance on the surface of friction pairs. Generally, extreme pressure agents are organic compounds containing S, P or Cl elements [11,12,13]. In recent years, compounds containing B elements have gradually been of concern for the sake of environmental protection [14,15]. Corrosion inhibitors can be employed to reduce the corrosion of copper foil caused by the rolling oil [16,17]. Organic heterocyclic compounds containing heteroatoms such as N, O and S have been used as corrosion inhibitors to hinder the corrosion on copper [16,17,18,19]. However, there is little research on what the main components are causing the corrosion of rolled copper foil and the corrosion mechanism of rolling oil on the rolled copper foil.

Therefore, static corrosion tests were carried out to study the corrosion of the main compositions in the rolling oil on the rolled copper foil in this work. The surface morphology and chemical composition of rolled copper foil after corrosion were analyzed by a digital camera, scanning electron microscope (SEM), energy dispersive spectrometer (EDS) and X-ray photoelectron spectroscopy (XPS) to investigate the corrosion mechanism of rolling oil on the rolled copper foil. The research results might provide a reference for selecting less corrosive additives used in the rolling oil and develop rolling oils with better performance.

## 2. Experimental

### 2.1. Corrosion Test

**Figure 1:**
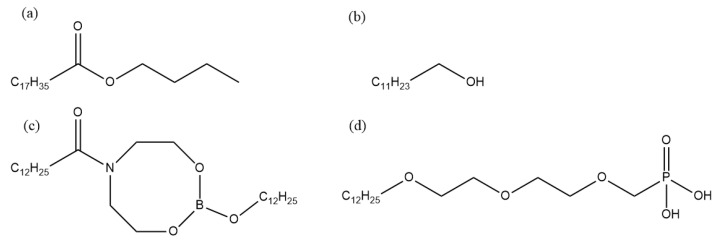
Molecular structures of different additives in the fully formulated rolling oil. (**a**) Butyl stearate, (**b**) dodecanol, (**c**) nitrogen-containing borate, (**d**) phosphate.

Static corrosion experiments were carried out on the rolled copper foil in order to explore corrosion characteristics of rolled copper foil in the rolling oil and main components in the rolling oil causing the corrosion of rolled copper foil. The corrosion media were the fully formulated rolling oil, the base stock and the base stock with only a kind of additive in the fully formulated rolling oil, respectively. In this paper, the white oil was selected as the base stock, butyl stearate and dodecanol were used as friction modifiers, and nitrogen-containing borate and phosphate were chosen as extreme pressure additives. The composition of different corrosive media was shown in Table 1, and molecular structures of additives in the fully formulated rolling oil was shown in Figure 1. The chemical composition of rolled copper foil (wt %) were shown as follows: Pb (0.0001), Si (<0.0004), Sb (<0.0002), Ni (<0.0003), Zn (<0.0009), Sn (<0.0002), Fe (<0.0009), P (<0.0008), Ag (<0.001), and Cu (balance).

The process of the static corrosion test was as follows: the rolled copper foil with a size of 50.00 × 50.00 × 0.18 mm was prepared. It was cleaned with acetone and carefully weighed three times by an electronic analytical balance after drying. The accuracy of the electronic analytical balance is 0.1 mg. The average value was taken as the initial weight of the rolled copper foil before the static corrosion test. The rolled copper foil was immersed in different corrosive media in beakers. Then, beakers with corrosive media and the rolled copper foil were put into an incubator. The temperature was set to 100 ℃. An experimental period was 3 h in this work. The rolled copper foil was taken out of the corrosive medium and put into acetone to prevent the air from oxidizing the rolled copper foil after each experimental period. It would be cleaned with acetone when the temperature of the rolled copper foil was reduced to room temperature. The weight of samples was measured after drying. The corrosion rate of the rolled copper foil in static corrosion tests was calculated by Equation (1) [20]:

(1) $$R=\frac{8.76\times 10^{7}\times (M-M_{1})}{STD}$$

where *R* is the corrosion rate of the rolled copper foil, mm/a; *M* is the weight of the rolled copper foil before static corrosion tests, g; *M*_1_ is the weight of the rolled copper foil after static corrosion tests, g; *S* is the total area of the rolled copper foil, cm^2^; *T* is the corrosion time, h; and *D* is the density of the rolled copper foil, kg/m^3^.

### 2.2. Corrosion Products Analysis

Macro-morphology of the rolled copper foil surface before and after corrosion experiments was observed by a digital camera (PowerShot G7 X Mark II, Canon Corporation, Tokyo, Japan) in the natural light, and micro-morphology was analyzed by an SEM (ULTRA 55 SEM, Carl Zeiss AG, Jena, Germany), to investigate the influence of rolling oil on the surface of the rolled copper foil. EDS (INCA X-MAX 50 EDS, Oxford Instruments, Oxford, UK) and XPS (PHI Quantera Ⅱ, Ulvac-Phi Inc., Kanagawa, Japan) were used to analyze the composition of the corroded rolled copper foil, which provided a reference for studying the corrosion mechanism of rolling oil on the rolled copper foil. SEM and EDS analysis was conducted in the secondary electrons. Electron acceleration voltages of SEM and EDS analysis were 20.00 and 15.00 kV, respectively. The excitation source of XPS was Al Kα radiation and the source energy was 1486.6 eV.

## 3. Results and Discussion

### 3.1. Corrosion Rate

**Figure 2:**
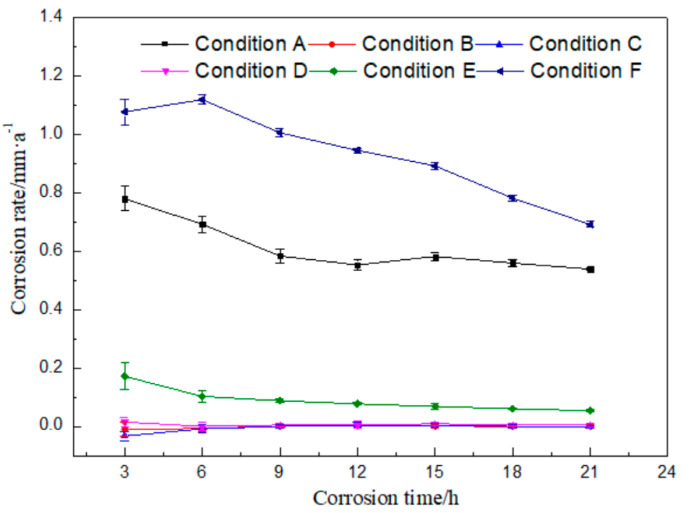
Corrosion rates of the rolled copper foil in different conditions against corrosion time.

There are plenty of components in the rolling oil. It is difficult to distinguish which kind of substances causes the corrosion of rolled copper foil, if static corrosion tests were done only in the fully formulated rolling oil. Therefore, static corrosion experiments were carried out on the rolled copper foil in the fully formulated rolling oil, the base stock and the base stock with only a kind of additive in the fully formulated rolling oil, respectively. The curve of corrosion rate with time was drawn according to the results of static corrosion experiments, as shown in Figure 2.

It could be seen from Figure 2 that the corrosion rate of rolled copper foil in condition A gradually reduced within 12 h, and there was only a small amount of fluctuation change after 12 h. The corrosion rate was the highest in the first 3 h, and then decreased with the increase of corrosion time in condition E. There was a slight increase for the corrosion rate of 6 h in condition F, compared with that of 3 h. Then, it decreased with the increase of corrosion time. Moreover, the corrosion rate of rolled copper foil in conditions B, C or D was very low, which was close to 0. The above experimental results indicated that there might be no obvious corrosion for the base stock and friction modifiers (butyl stearate and dodecanol) to the rolled copper foil, and it might be mainly the extreme pressure additives (N-containing borate and phosphate) in the rolling oil that caused the corrosion of rolled copper foil.

The general trend of the corrosion rate of rolled copper foil in conditions A, E or F was that it was relatively high in the initial test stage, and then gradually decreased. The reason for the above trend change might be that there were more effective corrosive media (N-containing borate or phosphate) in the rolling oil at the preliminary stage. They would be gradually consumed and the effective corrosive media in the rolling oil would reduce, as they corroded the rolled copper foil. It was difficult to cause a high corrosion rate to the rolled copper foil with relatively few corrosive media [21,22]. Therefore, the corrosion rate decreased with the increase of corrosion time.

The maximum corrosion rate of the rolled copper foil in condition E was obviously lower than that in condition F, which were about 0.17 and 1.12 mm/a, respectively. It manifested that phosphate was more corrosive than N-containing borate in the rolling oil. In addition, the maximum corrosion rate of the rolled copper foil in condition A was about 0.78 mm/a, which was lower than that in condition F. It indicated that there might be some components in the fully formulated rolling oil hindering the corrosion of phosphate to the rolled copper foil. N-containing borate and phosphate were the main compositions in the rolling oil causing the corrosion of rolled copper foil. It might also be the competition between N-containing borate and phosphate to the corrosion of the rolled copper foil, leading to a lower corrosion rate of phosphate in the fully formulated rolling oil.

### 3.2. Corrosion Products Analysis

**Figure 3:**
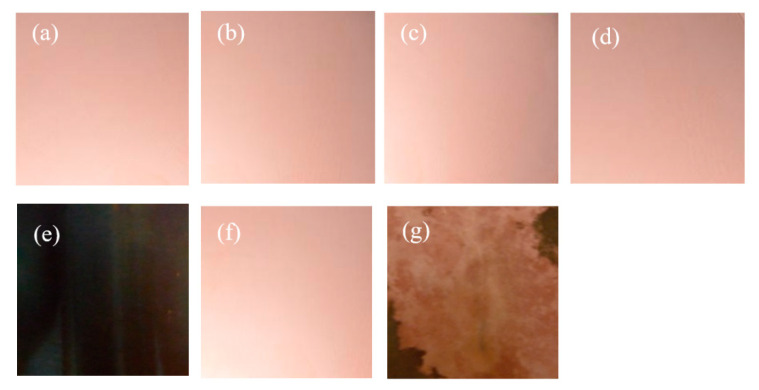
The original surface macro-morphology of the rolled copper foil (**a**) and its macro-morphology after corrosion in conditions B (**b**), C (**c**), D (**d**), E (**e**), F (**f**) and A (**g**).

The surface macroscopic morphology of the original rolled copper foil and its morphology after corrosion in conditions A to F were captured by a digital camera to investigate the influence of the corrosion of rolling oil on the rolled copper foil. The results are shown in Figure 3. It can be seen from Figure 3 that the color of the original rolled copper foil surface was pink (Figure 3a). It was nearly the same as the original rolled copper foil when corroded in conditions B, C, D or F (Figure 3b–d,f). The color of the rolled copper foil turned into purple-black when it was corroded in condition E (Figure 3e), while that of the rolled copper foil corroded in condition A was sophisticated. The corrosion characteristics of the rolled copper foil both in conditions E and F appeared on its surface. Most of the rolled copper foil surface was pink in different shades, and a few edge areas of the rolled copper foil was dark brown (Figure 3g). It demonstrated that the corrosion of the fully formulated rolling oil on the rolled copper foil might be the result of the synthetical effect of N-containing borate and phosphate.

**Figure 4:**
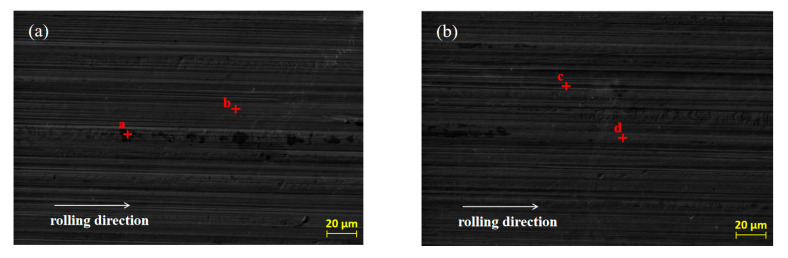

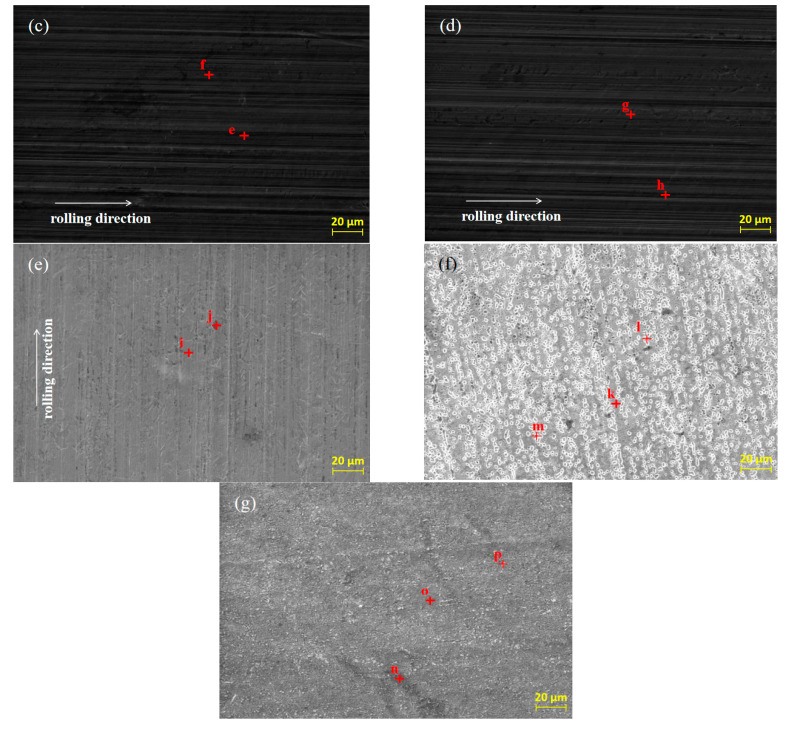
The original SEM morphology of the rolled copper foil (**a**) and its morphology after corrosion in conditions B (**b**), C (**c**), D (**d**), E (**e**), F (**f**) and A (**g**).

SEM was used to analyze the original micro-morphology of the rolled copper foil and the change of its micro-morphology after corrosion in conditions A to F, in order to further explore the influence of corrosion on the micro-morphology of the rolled copper foil surface. The results are shown in Figure 4.

As shown in Figure 4, both the original surface of the rolled copper foil and that corroded in conditions B, C, D and E were clean and the rolling texture on them was clear (Figure 4a–e). Comparing the above five types of rolled copper foil surface, the rolling texture of the corroded surface in condition E was shallower. However, the rolling texture on the surface of rolled copper foil after corrosion in condition F was nearly unrecognizable, and a large number of granular materials were uniformly distributed on the surface (Figure 4f). The rolling texture on the surface of rolled copper foil after corrosion in condition A was unclear, as well. There were three kinds of colors on the surface of rolled copper foil, which were black, gray and white, respectively. The black and gray areas were cross-distributed on the surface, while the white part was distributed on the gray area in a dotted way (Figure 4g). In order to investigate the corrosion of rolling oil on the rolled copper foil, an EDS was employed to analyze the chemical composition of point a to point p on the surface of the rolled copper foil in Figure 4, and the results of quantitative elemental analysis are shown in Table 2.

It could be concluded from Figure 4 and Table 2 that there was only Cu element at point b of the original rolled copper foil surface and there were also tiny amounts of C element at point a. The trace amounts of C element might result from the adsorption of cleaning agents before the EDS experiment or experimental error. Both the type and the quantity of elements of rolled copper foil corroded in conditions B, C or D were similar (Figure 4b–d; point c to h in Table 2). They were composed of more than 98 wt % Cu element and a small amount of C element. The C element on the tested rolled copper foil might be caused by the adsorption of hydrocarbon molecules in the base stock on the surface of the corroded rolled copper foil [23]. The composition of point i on the rolled copper foil corroded by condition E (point i in Figure 4e) was B, C, N, O and Cu elements, while that of point j (point j in Figure 4e) was mainly C, O and Cu elements. The O element on the surface of the corroded rolled copper foil indicated that it was oxidized in condition E, which was consistent with the blackening color of the rolled copper foil surface (Figure 3e). There might be some hydrocarbon molecules adsorbed on the surface of the corroded rolled copper foil resulting in C element on the corroded surface [23]. The B and N elements contained on the surface of rolled copper foil might mainly come from the adsorbed N-containing borate or the reaction products of it. The shallow rolling texture on the corroded surface of rolled copper foil might be due to the chemical polishing effect caused by the reaction between the N-containing borate and the surface of rolled copper foil [24,25,26]. There was more copper in the higher part that reacted with the N-containing borate and dissolved in the corrosive medium and/or a thicker oxidation product was produced on the lower part. Oxidation products formed on the surface of rolled copper foil could slow down the corrosion of N-containing borate to a certain extent [27,28]. Therefore, the corrosion rate of rolled copper foil decreased with the increase of corrosion time, owing to the formation of oxidation products (Figure 2).

There were only C and Cu elements on the main part (point k in Figure 4f), the granular part (point m in Figure 4f) and the boundary position of above two parts (point n in Figure 4f) of the rolled copper foil surface after corrosion in condition F. It demonstrated that copper might be changed into copper ion or organic compound through chemical reaction between copper and the phosphate, which was soluble in the base stock [29,30]. They would be dissolved in the base stock, and the inner copper layer was exposed to the surface. This was consistent with the pink color of the rolled copper foil surface after corrosion in Figure 3f. A certain amount of hydrocarbon molecules might be adsorbed on the fresh surface. Therefore, there was a C element on the corroded surface. The composition of different parts of the corroded surface of rolled copper foil was generally consistent. The spot particles formed on the corroded surface (Figure 4f) might be due to the uneven reaction between the rolled copper foil and phosphate.

The black part on the surface of rolled copper foil corroded in condition A (point n in Figure 4g) was composed of C, O, N and Cu elements. The gray part (point o in Figure 4g) mainly contains C, O and Cu elements, while there were only C and Cu elements in the white part (point p in Figure 4g). It manifested that the corrosion of rolled copper foil surface in the fully formulated rolling oil was different. The white part might mainly be corroded by phosphate, and corrosion products were dissolved in the rolling oil with the fresh copper exposed to the surface. The gray and black parts might mainly be corroded by N-containing borate, and they were mainly composed of oxidation products of copper. There might be some N-containing borate and/or its reaction products adsorbed on the black part. Therefore, it also contained some N elements. There might be competition between N-containing borate and phosphate on the corrosion of rolled copper foil, leading to the uneven corrosion of rolled copper foil surface and a lower corrosion rate comparing with that in condition F (Figure 2).

**Figure 5:**
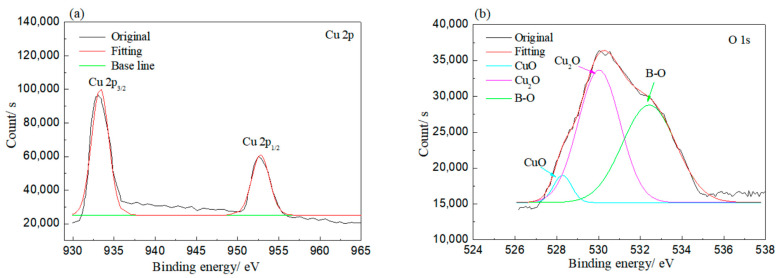

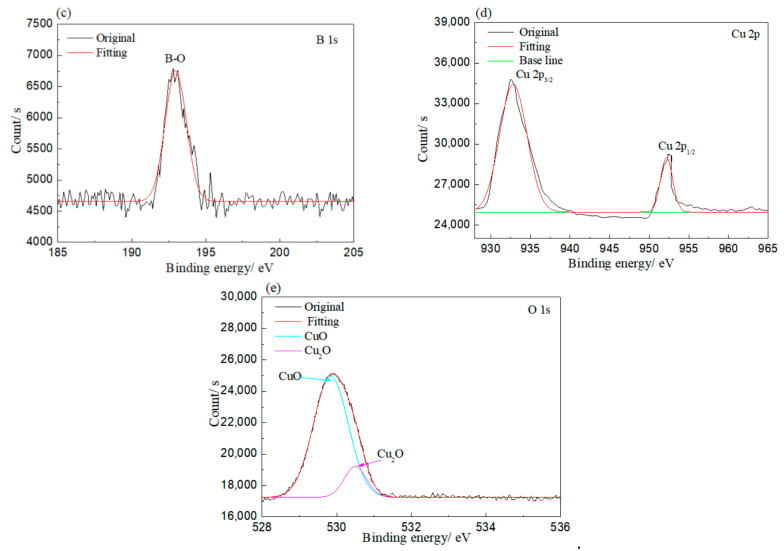
XPS results of rolled copper foil corroded in condition E (**a**–**c**); and condition A (**d**,**e**).

XPS was employed to investigate the bonding of elements on the corroded rolled copper foil, and the results are shown in Figure 5. As shown in Figure 5, Cu and O elements were detected on the rolled copper foil corroded in both condition E and condition A. Peaks at 930–935 and 950–955 eV in Figure 5a and peaks at 528–532 and 527–529 eV in Figure 5b indicated that the Cu element corroded in condition E might be in the state of Cu, CuO and/or Cu_2_O, while the O element was in the state of CuO and/or Cu_2_O [31]. The O element might also be bonded to the B element according to the peak at 530–535 eV in Figure 5b and the peak at 192–195 eV in Figure 5c [31]. It might come from adsorbed N-containing borate or its reaction products. Peaks corresponding to the Cu element in Cu, CuO and/or Cu_2_O and peaks related to the O element in CuO and/or Cu_2_O were found in the XPS result of rolled copper foil corroded in condition A as well (Figure 5d,e). However, the B–O bond was not detected. It could be concluded from the above analysis that the rolled copper foil was oxidized into CuO and/or Cu_2_O in both condition E and condition A. N-containing borate or its reaction products were adsorbed on the surface of rolled copper foil in condition E.

### 3.3. Corrosion Mechanism

**Figure 6:**
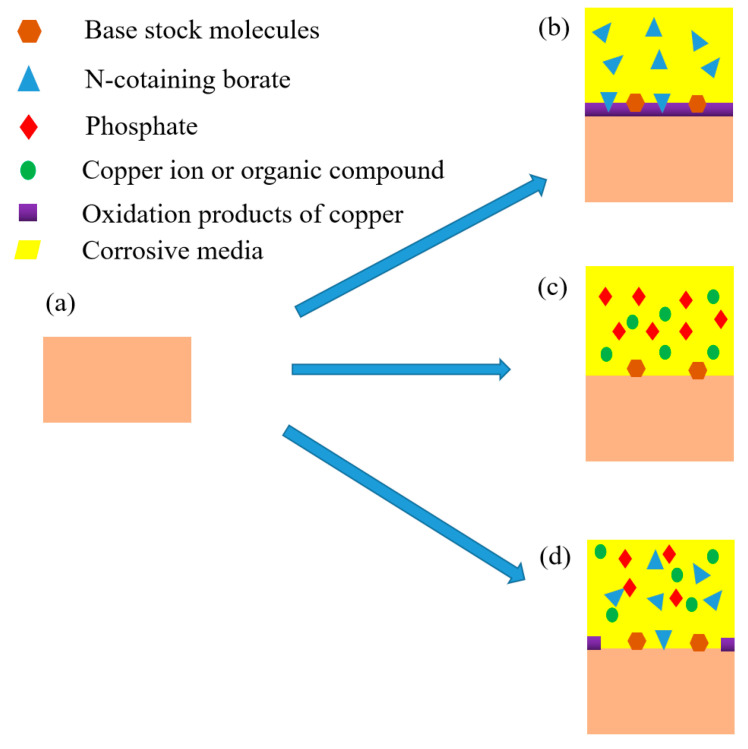
The original rolled copper foil (**a**) and sketches of the corroded rolled copper foil in condition E, (**b**) F (**c**) and A (**d**).

According to above analysis, the corrosion mechanism of rolled copper foil in various corrosive media were different. Sketches of the corrosion process of rolled copper foil in conditions A, E and F were shown in Figure 6. As shown in Figure 6b, a layer of copper oxide film was formed on the surface of rolled copper foil when corroded in condition E. This film could protect the inner copper from contacting with corrosive N-containing borate, and thus reduce the corrosion rate [27,28]. It might be the reason for the relatively low corrosion rate of rolled copper foil in condition E (Figure 2). Some N-containing borate and base stock molecules could adsorb on the surface of rolled copper foil due to the van der Waals force between them. Phosphate mainly reacted with copper to soluble substances in condition F, and some base stock molecules were adsorbed on the inner surface of corroded rolled copper foil (Figure 6c). The fresh copper surface would continue to react with phosphate, leading to a relatively high corrosion rate (Figure 2). Both phosphate and N-containing borate could react with the rolled copper foil. Competing reactions of them might reduce the corrosion rate of rolled copper foil in the fully formulated rolling oil. Moreover, N-containing borate could react with the rolled copper foil to oxidation products of copper in some areas of the rolled copper foil surface. This protective film would prevent the corrosion of N-containing borate and phosphate on the rolled copper foil (Figure 6d), and decrease the corrosion rate. It might be the reason for why the corrosion rate of rolled copper foil in condition A was lower than that in condition F (Figure 2).

## 4. Conclusions

In this paper, the corrosion of various components in the rolling oil on the rolled copper foil was studied by static corrosion experiments. The corrosion rate, the surface morphology and chemical compositions of the corroded rolled copper foil were analyzed. The following conclusions were drawn.

The corrosion rate of rolled copper foil in the base stock and friction modifiers (butyl stearate, dodecanol) was quite low. It might be mainly extreme pressure additives (N-containing borate and phosphate) that caused the corrosion of rolled copper foil. The corrosion rate of rolled copper foil generally decreased with the increase of corrosion time in the N-containing borate, phosphate and the fully formulated rolling oil.

N-containing borate could react with the rolled copper foil to form oxidation products of copper (CuO and/or Cu_2_O), resulting in the darkening of the surface color of the rolled copper foil. The oxidation products could protect the inner copper foil from corrosion. N-containing borate or its reaction products might adsorb on the rolled copper foil. The reaction between phosphate and the rolled copper foil mainly produced substances that could be dissolved in the rolling oil. The fresh copper surface would further be corroded, which lead to a relatively large corrosion rate of rolled copper foil.

There might be competition between N-containing borate and phosphate on the corrosion of rolled copper foil in the fully formulated rolling oil. In addition, protective films might be formed on the surface of rolled copper foil. Therefore, the corrosion rate of rolled copper foil in the fully formulated rolling oil was lower than that in the phosphate.

## Figures and Tables

**Table 1:** Compositions of different testing conditions, the fully formulated rolling oil (A), the base stock (B), and the base stock containing only dodecanol (C), butyl stearate (D), Nitrogen-containing borate (E) or phosphate (F).

Conditions	Base Stock/g	Dodecanol/g	Butyl Stearate/g	Nitrogen-Containing Borate/g	Phosphate/g
A	95.5	1.0	1.5	0.5	1.5
B	95.5	–	–	–	–
C	95.5	1.0	–	–	–
D	95.5	–	1.5	–	–
E	95.5	–	–	0.5	–
F	95.5	–	–	–	1.5

**Table 2:** Quantitative elemental analysis of the original rolled copper foil surface (a),(b) and corrosion products of the rolled copper foil surface corroded in condition B (c),(d); condition C (e), (f); condition D (g), (h); condition E (i), (j); condition F (k), (l), (m) and condition A (n), (o), (p).

Number	Content/Error	Cu	C	O	B	N
a	Content/wt %	99.38	0.62	–	–	–
	Error/wt %	4.6	0.4	–	–	–
b	Content/wt %	100	–	–	–	–
	Error/wt %	4.7	–	–	–	–
c	Content/wt %	98.04	1.96	–	–	–
	Error/wt %	4.4	0.8	–	–	–
d	Content/wt %	98.32	1.68	–	–	–
	Error/wt %	4.6	0.7	–	–	–
e	Content/wt %	98.45	1.55	–	–	–
	Error/wt %	4.5	0.7	–	–	–
f	Content/wt %	98.17	1.83	–	–	–
	Error/wt %	4.4	0.8	–	–	–
g	Content/wt %	98.33	1.67	–	–	–
	Error/wt %	4.7	0.7	–	–	–
h	Content/wt %	98.28	1.72	–	–	–
	Error/wt %	4.5	0.7	–	–	–
i	Content/wt %	81.75	9.77	2.73	5.26	0.49
	Error/wt %	3.7	2.6	0.7	2.3	0.3
j	Content/wt %	85.06	7.96	6.98	–	–
	Error/wt %	3.6	2.4	1.7	–	–
k	Content/wt %	98.22	1.78	–	–	–
	Error/wt %	4.4	0.8	–	–	–
l	Content/wt %	98.16	1.84	–	–	–
	Error/wt %	4.4	0.8	–	–	–
m	Content/wt %	97.87	2.13	–	–	–
	Error/wt %	4.7	1.0	–	–	–
n	Content/wt %	89.44	7.96	1.77	–	0.83
	Error/wt %	4.2	2.1	0.4	–	0.5
o	Content/wt %	91.55	6.16	2.29	–	–
	Error/wt %	4.1	1.7	0.7	–	–
p	Content/wt %	95.43	4.57	–	–	–
	Error/wt %	4.4	1.2	–	–	–
